# A Comparative Efficacy of Low-Dose Combined Oral Contraceptives Containing Desogestrel and Drospirenone in Premenstrual Symptoms

**DOI:** 10.1155/2013/487143

**Published:** 2013-02-20

**Authors:** Jirath Wichianpitaya, Surasak Taneepanichskul

**Affiliations:** Family Planning Unit, Reproductive Medicine Division, Department of Obstetrics and Gynecology, Faculty of Medicine, Chulalongkorn University, Rama IV Road, Bangkok 10330, Thailand

## Abstract

*Objective*. To compare the efficacy of low-dose COC containing desogestrel (DSG) with drospirenone (DRSP) in the changes of premenstrual symptoms. *Methods*. In an open-label randomized controlled trial, 90 women with premenstrual syndrome who required COC were randomly recruited and allocated equally to receive either 6 cycles of 20 micrograms ethinyl estradiol (EE)/150 micrograms DSG (DSG group) or 20 micrograms EE/3 mg DRSP (DRSP group) in 24/4 extended regimen. Analysis of covariance and repeated analysis of variance were used to determine the difference of mean Women's Health Assessment Questionnaire (WHAQ) scores changes between groups, within group, and in premenstrual, menstrual, and postmenstrual phases. *Results*. Baseline characteristics and WHAQ scores were comparable. At the ends of the 3rd and the 6th cycles, mean WHAQ scores of all the 3 phases in DRSP group showed significant reduction and were significantly lower than those in DSG group. DSG group showed significant reduction in both premenstrual and menstrual phases after the 6th cycle. Adverse effects were comparable in both groups. In conclusion, low-dose COC containing either DSG or DRSP reduced premenstrual symptoms, but the latter showed greater efficacy and earlier reduction.

## 1. Introduction

About 20%–90% of women experience premenstrual syn-drome (PMS), and 2%–15% of them have severe symptoms [1]. Among Thai females at Family Planning Unit, KingChulalongkorn Memorial Hospital, 56% of them have PMS [2]. PMS is a common problem with different levels of severity (Table 1). Possible mechanisms include hormonal influences on the central nervous system; mood symptoms may reflect hormonal changes. A randomized crossover trial reported that women with PMS had an abnormal response to normal concentrations of gonadal steroids. The current research areas include neurotransmitters, especially serotonin and gamma-aminobutyric acid (GABA), which can affect mood and behavior [3–5].

Criteria for PMS were developed in the 1980s [6]. According to O'Brien [7], Women's Health Assessment Questionnaire (WHAQ), which was a subset of items selected from the Menstrual Distress Questionnaire (MDQ), can be used in assessment PMS. 

 Modifying the 7-day hormone-free interval (HFI) has also been investigated and is currently beginning to appear in COCs. A 24/4 day regimen of low-dose COC containing drospirenone (DRSP) regimen which reduces HFI has been investigated and approved by U.S. Food and Drug Administration [8, 9]. This regimen was more effective than placebo in reducing premenstrual symptoms and improving the quality of life in women with PMDD [10].

20 *μ*g ethinyl estradiol (EE)/150 *μ*g desogestrel (DSG) preparation provided good cycle control with less treatment discontinuation due to the acceptable bleeding pattern and the decrease in the severity of PMS [11]. The shortened HFI has been designed to provide greater suppression of follicle development and a more stable level of exogenous hormones throughout the menstrual cycle, thereby reducing adverse symptoms during the HFI [12]. It has been developed for patients who experience significant withdrawal symptoms [13].

So there is suspicion whether the former low-dose COC would be modified in terms of extended 24/4 regimen in order to comparably relieve the premenstrual symptoms [14]. The purpose of this study was to compare the efficacy of low-dose COC containing 15 *μ*g DSG with 3 mg DRSP in premenstrual symptoms.

## 2. Material and Methods

### 2.1. Study Design and Population

An open-label randomized controlled trial was performed at the Family Planning Clinic, King Chulalongkorn Memorial Hospital from June 2011 to February 2012.

Child bearing-aged women who were requesting COCs for contraception were recruited from many ways including advertisement, invitation to postpartum and postabortion visits and inviting some who had appointments with the Clinic.

All subjects who met the eligible criteria were considered to participate in the study.

The inclusion criteria consisted of women aged 18–35 years had regular menstrual cycles lasting between 21 and 35 days, did not use injectable or implant hormonal contraceptives within 6 months or oral contraception and IUD within 3 months before the study, and did not wish to use any other form of hormonal treatment, including other hormonal contraception, for the duration of the study protocol. Postabortion and postpartum patients were eligible if they had had three consecutive menstrual cycles before the study.

The exclusion criteria included women who were pregnant or suspected of being pregnant, who were breastfeeding, who were smoking, who were contraindicated for using COCs according to WHO categories 2, 3, and 4, and finally who had PMDD.

### 2.2. Study Protocol and Data Collection

**Figure 1:**
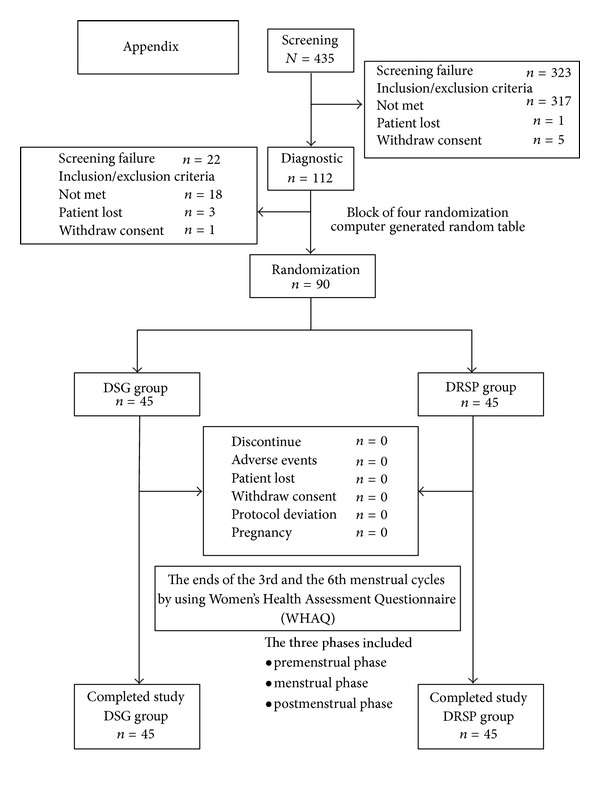
Participation flow. DSG group used the preparation of ethinyl estradiol 20 *μ*g/desogestrel 150 *μ*g, and DRSP group used the preparation of ethinyl estradiol 20 *μ*g/drospirenone 3 mg.

The study protocol including participant flow as shown in Figure 1 was approved by the Institutional Review Board, Faculty of Medicine, Chulalongkorn University. To meet the criteria of PMS diagnosis according to the ACOG 2000, a patient had to report at least one of the following affective symptoms and to report at least one of the following somatic symptoms during the 5 days before menses in each of the 3 prior menstrual cycles.

The symptoms had to be relieved within 4 days of menstruation onset without recurrence until at least the cycle day 13th. The symptoms usually present in the absence of the following:

1. pharmacologic therapy,
2. hormone ingestion,
3. drug,
4. alcohol abuse.

Patients suffered from identifiable dysfunction in social or economic performance.

All subjects were required to sign written informed consent forms before being recruited. Randomization numbers were generated in fixed block of four and were allocated in 1 : 1 ratio. The women underwent general physical and pelvic examinations, along with the cervical Pap test—if no previous 1 year examination had been done, and had urinary pregnancy test at the screening and diagnostic period in the initial step of protocol. All subjects were randomly assigned to two groups: group 1 (DSG group) which used the preparation of ethinyl estradiol 20 *μ*g/desogestrel 150 *μ*g and the other group 2 (DRSP group) which used the preparation of ethinyl estradiol 2 *μ*g/drospirenone 3 mg. Randomized subjects were asked to start the COC on the 1st day of menstrual cycle and continue until the 24th day. The last 4 days of the pill pack consisted of inert placebo pills for the DRSP group 2, and for the DSG group 2, the subjects would be informed not to take any pills for 4 days. The analysis of outcomes at the end of each treatment cycle by independent statistical analyst and the results were also checked by the investigators. The results were determined for potential errors before presentation in the department and the institutional progression review. 

The subjects were asked to fill the baseline characteristic demographic data in the form and submit it to the nurse coordinator. Vital signs, body weight, body height, and body mass index (BMI) were collected and calculated.

Data were collected at the main visits by assistant nurses and scientists who were blinded from the protocol at the Family Planning Clinic, King Chulalongkorn Memorial Hospital. Arranged data were collected on the visits consistent with the protocol consisted of the following:

- baseline—before the COCs use,
- the second visit—after completed 3 cycles use of COCs,
- the last visit—after completed 6 cycles use of COCs.

Both groups of subjects were evaluated on the premenstrual symptoms at baseline and at the ends of the 3rd and the 6th cycles by using Women's Health Assessment questionnaire (WHAQ) which was prepared in self-report form. WHAQ was a subset of items selected from the Menstrual Distress Questionnaire. WHAQ was translated from English to Thai and tested for reliability with a Cronbach's alpha of 80. We were allowed for using this version. Three phases included the following:

- premenstrual phase: the 4-day period before menstruation,
- menstrual phase: the first day through the last day of menstruation
- postmenstrual phase: the reminder of the cycle.

This self-report questionnaire included 6 categories that contained 23 items. The 6 categories were composed of 3 main categories that included

1. impaired concentration,
2. water retention,
3. negative affect.

Another 3 additional categories included

1. increased appetite,
2. feelings of well-being,
3. undesirable hair changes.

The impaired concentration category contained eight items. The water retention category contained four items, and the category of negative effect contained eight items, as shown in Table 2. Each item was graded using a five-point scale from 0 to 4 (0 = absent, 1 = mild, 2 = moderate, 3 = strong, and 4 = severe/very strong).

In addition, adverse events that occurred were reported and managed as soon as possible. The packages of COCs were checked for potential medication damage or medication expiration and also were checked for compliance. All subjects were informed about their right to discontinue the participation in the project.

Adverse drug reactions (ADRs) were evaluated at the end of the 3rd cycle and at the end of the 6th cycle. The subjects were able to report ADRs as they were faced as soon as possible by telephone. 

### 2.3. Statistical Analysis

Data were analyzed from intent-to-treat analysis in which randomized subjects included who took at least 1 cycle of COC. Per-protocol analysis would be determined in comparing the outcomes in cases of discontinuation were found.

Repeated analysis of variance (repeated ANOVA) was used to determine the mean WHAQ scores changes within each of the treatment groups.

Analysis of covariance (ANCOVA) was used to determine the difference between the two treatment groups. ANCOVA contained the term of baseline WHAQ score as a covariate. 

Baseline characteristic demographic data were determined using descriptive statistics and were compared between the two groups using independent *t*-test according to continuous data or Chi-square test according to categorical data. 

Level of significance was considered at 95 percent confidence interval and *P* value less than 0.05. Statistical Package for Social Sciences (SPSS) program for Windows version 17 which had been allowed for permitted uses in academic studies was used for statistical analyses.

## 3. Results

There were a total of 90 subjects allocated to either group in 1 : 1 ratio. All of them had completed the protocol, although 5 of them had temporary losses but did not affect the protocol. The reason of the losses was resulting from devastating flood crisis in the middle part of Thailand including, but not all, part of Bangkok. There was no statistically significant differences in baseline characteristic demographic data between both groups as shown in Table 3, including age, height, body weight, body mass index, blood pressure, parity, and education level.

According to Table 4, baseline WHAQ scores were comparable. The common premenstrual symptoms included negative effect, water retention, and impaired concentration accordingly. As expected before, the most severe symptoms occurred at the premenstrual phase rather than the menstrual phase and were almost negligible in the postmenstrual phase. There was no reported undesirable hair changes symptom according to WHAQ. 

 At the end of the 3rd menstrual cycle, mean WHAQ scores in the DRSP group were significantly lower than those DSG group in all the 3 phases of menstrual cycle; even the baseline WHAQ score in DRSP group was a little nonsignificantly higher. Predominately, at the end of the 6th menstrual cycle, mean WHAQ scores in DRSP group were continuing to decrease significantly much more than those the DSG group. 

Based on Table 5, reduction effect resulted from intervention was showed regarding premenstrual symptoms. Except for the marginal reduction effect in postmenstrual phase determined between the 3rd and the 6th cycles, DRSP group showed significant reduction in mean WHAQ scores of all the three phases at the 3rd and the 6th cycles, while DSG group showed significant reduction in menstrual phase and premenstrual phase at the 6th cycle between the 3rd and the 6th cycles and postmenstrual phase between the 3rd and the 6th cycles. In the subgroup analysis of DSG group, there were reduction effects of negative effect and impaired concentration at the ends of the 3rd and the 6th cycles and partial reduction effect of water retention in phases of premenstrual symptoms. In contrast, DRSP group showed almost all the 3 phases with reduction effect of the common premenstrual symptoms along the protocol. As shown in Tables 4 and 5, among the uncommon premenstrual symptoms, there appeared negligible effects of the intervention from both groups.

 Adverse effects were identified in Table 6. Adverse effects were negligible including nausea, dizziness, amenorrhea, vaginal spotting, and mastalgia. The adverse effects were comparable in both groups. 

Repeated ANOVA showed significant body-weight reduction at the end of 6th cycle and between the 3rd and the 6th cycles in DRSP group, but there was no significant change in body-weight along the protocol in DSG group. According to Table 7, Using ANCOVA model, body weight change was different between groups significantly at the end of the 6th but not of the 3rd cycle. 

No serious adverse drug effects and inadvertent pregnancy were reported.

## 4. Discussion

WHAQ score could range from 0 to the maximal score of 92. WHAQ was a subset of items selected from the Menstrual Distress Questionnaire [7]. WHAQ was a questionnaire translated from English to Thai and tested for reliability with a Cronbach's alpha of 80. This tool had been used as an assessment tool of premenstrual symptoms in response to several interventions [15, 16].

 This study demonstrated the main result in which low-dose COC containing DRSP reduced the premenstrual symptoms as measured by WHAQ, and it provides good contraceptive efficacy consistent with the previous studies [17, 18].

Although the etiology of the premenstrual disorders has not been understood, the symptoms seem to occur primarily in the setting of ovulatory menstrual cycles and have been hypothesized to result from alterations between gonadal hormones and CNS neurotransmitters including serotonin, GABA, and endorphins, as well as other modulators, in genetically predisposed individuals [19, 20]. Anovulation should abolish premenstrual symptoms, and oophorectomy and medical menopause are effective therapeutic modalities for severe PMS. However, simply providing an anovulatory condition using a COC was not generally found to be effective for the treatment of PMS symptoms [21].

Some authors supposed that rise and fall of sex steroids can precipitate premenstrual symptoms. Even more, the hormone-free interval resulted from the formulation of 21/7 COC would contribute to the aggravating symptoms. So the shortening of the HFI to 3-4 days was proposed in order to maintain sufficient levels of exogenous estrogen and progestin to inhibit follicular development and suppress ovarian steroids synthesis [22]. 

 The newer OC formulation of DRSP/EE 3 mg/20 *μ*g (24/4) is an effective contraception that is well tolerated, with favorable bleeding profile [19]. A large, multicenter randomized controlled trial investigates the effect on symptoms resulted from PMDD. In a parallel-group study of this formulation of DRSP/EE 3 mg/20 *μ*g (24/4), there was a 50% decrease in PMDD symptoms in 48% of women ingesting active drug versus 36% with placebo, and a statistically significant difference appeared after 3 cycles [23]. According to cycle-cycle variability of PMS, we decided to study in a longer intervention period (i.e., 6 cycles) regarding the effect of the low-dose COC formulation of DRSP/EE 3 mg/20 *μ*g (24/4) on premenstrual symptoms.

Using ANCOVA model which limits the potential errors resulting from uneven baseline parameters between groups, even at the baseline, self-report showed nonsignificant higher mean WHAQ score, and low-dose COC containing DRSP seemed to be more efficient than low-dose COC containing DSG regarding the premenstrual symptoms along the study period of 6 cycles.

DRSP is pharmacologically similar to endogenous progesterone. Progesterone is known to exert direct sedative effect on the central nervous system, as reflected in the slowing of electroencephalogram in humans and the alteration of arousal threshold stimuli in the hypothalamus of various animals. Deficiency of progesterone would increase nervous excitability, irritability, tension, anxiety, and aggression [24]. Therefore, the possible reason of the benefits of the negative effect of DRSP may be due to the chemical structure similarity of DRSP to progesterone in these effects rather than DSG [25].

Some investigators have demonstrated abnormally elevated plasma testosterone in women with premenstrual symptoms and aggression [26]. Therefore, it was possible that the improvement of the negative effect in women who were receiving DRSP compared to those receiving DSG was related to the antiandrogenic property of DRSP. Given that DRSP does not bind to sex hormone binding globulin (SHBG), it may not allow the rising of serum-free testosterone that might be accounted to aggravation of premenstrual symptoms and aggression. Importantly, the effect of EE effect on the increasing of SHBG was not negatively influenced by DRSP [27]. 

Unlike the other kinds of progestin, DRSP has antimineralocorticoid (aldosterone antagonistic) and anti-androgenic properties [28]. Also, the structure of DRSP is similar to a diuretic antihypertensive drug named spironolactone. Overall, those pharmacological properties of DRSP might explain the results in our study in accordance with showing of stronger effect of on premenstrual symptoms. 

Also, our study showed that low-dose COC containing DSG in an extended 24/4 regimen has an effect on the premenstrual symptoms regarding the severity of reduction. This result showed one of the noncontraceptive benefits which might be due to lower estrogen (EE) dose, the effect of decreasing HFI and probably the minimal androgenic effect of DSG among gonane subgroups within the 19-nortestosterone group of progestogens.

The other potential strength of the study was the lacking of discontinuation, since the study recruitment reflected in good pills compliance which was shown in the follow-up visits. In the study, beside the premenstrual symptoms reduction effect, either of COCs showed favorable body-weight changes and comparable minimal events of adverse effects. Those might explain the good compliance in both DSG and DRSP groups. The trend towards a slight decrease in body weight in DRSP group was consistent with the previous study using DRSP containing COC [20].

There are some possible limitations of this study. First, an open-label randomized controlled trial may have influence on self-report regarding the premenstrual symptoms. Second, the main outcome and the average WHAQ scores were rather subjective and based on the recent memory that might be affected by several factors.

The premenstrual symptoms which are partition of PMS have been considered as an individual persistent problem among general childbearing-aged women. These cyclic symptoms have repeatedly affected their physical, emotional, and psychological well being resulting in a negative impact on the quality of life, or even, the surrounding people. Several studies have been performed to accomplish the discovery of medications that can improve these symptoms. Given the positive study results from either DSG or DRSP group, it was the potential benefits of the study included characteristics of noncontraceptive efficacy, especially the effect on PMS, that resulted from COCs in an extended regimen use. 

To our knowledge, this study was the first study which compared the efficacy of low-dose COC formulations containing either DRSP or DSG regarding the premenstrual symptoms. Until more data from sufficient studies demonstrate the therapeutic efficacy according to other kinds of modalities or other COCs, either of COCs used in this study may be an alternative choice for the selected women seeking contraception who suffer from some degree of PMS. The future studies may have objectives to investigate the therapeutic effects of other extended COC regimen, in order to minimize or abolish HFI or other potential COCs in the selected population with premenstrual symptoms. 

In conclusion, from our study, low-dose COC containing either DSG or DRSP could reduce the premenstrual symptoms. Low-dose COC containing DRSP showed greater efficacy and earlier reduction regarding the premenstrual symptoms than low-dose COC containing DSG according to the interval period of either 3 or 6 cycles.

## Figures and Tables

**Table 1:** Group of symptoms according to ACOG diagnostic criteria.

Somatic symptoms	Effective symptoms
(i) Breast tenderness (ii) Abdominal bloating (iii) Headache (iv) Swelling of extremities	(i) Depression (ii) Angry outbursts (iii) Irritability (iv) Anxiety (v) Confusion (vi) Social withdrawal

**Table 2:** Categories (6) and items (23) in each main category of WHAQ scores.

Negative effect (8 items)	Water retention (4 items)	Impaired concentration (8 items)	Additional categories (3 items)
(i) Loneliness (ii) Anxiety (iii) Mood swings (iv) Crying (v) Irritability (vi) Tension (vii) Feeling sad or blue (viii) Restlessness	(i) Weight gain (ii) Painful or tender breasts (iii) Breast and abdominal swelling (iv) Skin blemishes or disorders	(i) Insomnia (ii) Forgetfulness (iii) Confusion (iv) Poor judgment (v) Difficulty in concentrating (vi) Distractibility (vii) Poor motor coordination (viii) Minor accidents	(i) Increased appetite (ii) Feelings of well being (iii) Undesirable hair changes

**Table 3:** Baseline characteristic demographic data.

Characteristics	Treatment group	
	DSG group (*n* = 45)	DRSP group (*n* = 45)
Age (year ± SD)	26.6 (±3.9)	27.9 (±3.4)
BW (kg ± SD)	55.6 (±7.9)	56.5 (±8.4)
Height (cm ± SD)	157.1 (±5.2)	158.9 (±6.1)
BMI (kg/m^2^ ± SD)	22.5 (±2.9)	22.4 (±3.0)
Parity (median ± range)	1 (0–4)	1 (0–4)
Education level		
primary	7	2
secondary	24	24
graduated	13	8
postgraduated	1	1

**Table 4:** The results of group and subgroup analyses according to the comparison of mean WHAQ scores changes between groups.

		Between groups								
ANCOVA model										
		Premenstrual			Menstrual			Postmenstrual		
Mean WHAQ scores		DSG	DRSP	Sig	DSG	DRSP	Sig	DSG	DRSP	Sig
	Baseline	19.49 ± 6.76	21.33 ± 8.53	0.259	17.13 ± 5.88	17.42 ± 6.41	0.824	5.84 ± 1.98	6.16 ± 2.88	0.552
Total	3rd cycle	19.07 ± 6.27	14.78 ± 6.28	0.000	17.82 ± 6.11	12.98 ± 4.27	0.000	5.96 ± 2.04	4.80 ± 1.50	0.000
	6th cycle	16.24 ± 6.48	7.62 ± 2.46	0.000	15.40 ± 5.42	7.49 ± 2.87	0.000	5.38 ± 1.54	4.29 ± 0.82	0.000
	Baseline	7.76 ± 3.21	7.38 ± 3.77	0.610	7.16 ± 3.02	5.93 ± 3.20	0.066	1.51 ± 1.36	1.38 ± 1.48	0.657
Negative effect	3rd cycle	7.33 ± 2.95	5.07 ± 3.13	0.000	7.11 ± 2.99	4.36 ± 2.68	0.000	1.47 ± 1.38	0.80 ± 1.14	0.006
	6th cycle	5.60 ± 3.10	1.56 ± 1.52	0.000	5.67 ± 2.99	1.58 ± 1.34	0.000	1.13 ± 1.27	0.07 ± 0.33	0.000
	Baseline	4.71 ± 3.08	5.51 ± 3.61	0.261	2.87 ± 2.74	3.62 ± 2.90	0.208	0.36 ± 0.80	0.69 ± 1.18	0.121
Water retention	3rd cycle	4.51 ± 2.98	3.64 ± 2.39	0.000	3.51 ± 2.67	2.42 ± 1.83	0.000	0.56 ± 0.94	0.29 ± 0.66	0.005
	6th cycle	3.87 ± 2.37	1.98 ± 1.82	0.000	3.22 ± 2.32	1.33 ± 1.43	0.000	0.38 ± 0.61	0.16 ± 0.56	0.043
	Baseline	4.49 ± 3.29	5.84 ± 4.31	0.097	4.56 ± 3.17	5.27 ± 3.60	0.322	0.73 ± 0.96	0.87 ± 1.12	0.546
Impaired concentration	3rd cycle	4.11 ± 3.41	3.27 ± 3.11	0.000	3.96 ± 2.90	3.02 ± 2.34	0.000	0.62 ± 0.94	0.36 ± 0.68	0.063
	6th cycle	3.27 ± 3.14	0.93 ± 1.10	0.000	3.20 ± 2.70	1.11 ± 1.49	0.000	0.38 ± 0.86	0.04 ± 0.30	0.013
	Baseline	1.84 ± 1.63	2.09 ± 1.47	0.457	1.53 ± 1.34	1.80 ± 1.29	0.339	0.18 ± 0.54	0.27 ± 0.58	0.452
Increased appetite	3rd cycle	1.96 ± 1.36	1.33 ± 1.07	0.000	1.58 ± 1.20	1.11 ± 0.96	0.000	0.36 ± 0.57	0.11 ± 0.32	0.007
	6th cycle	2.04 ± 1.28	1.07 ± 1.03	0.000	1.38 ± 1.15	0.62 ± 0.86	0.000	0.24 ± 0.48	0.11 ± 0.38	0.167
	Baseline	0.64 ± 0.83	0.51 ± 0.63	0.392	1.04 ± 0.85	0.80 ± 0.79	0.161	3.02 ± 0.66	2.96 ± 0.64	0.626
Feeling of well being	3rd cycle	1.11 ± 0.91	1.47 ± 0.73	0.008	1.62 ± 0.75	2.07 ± 1.01	0.002	2.91 ± 0.76	3.24 ± 0.86	0.042
	6th cycle	1.42 ± 0.81	2.09 ± 0.67	0.000	1.89 ± 0.83	2.78 ± 0.67	0.000	3.20 ± 0.76	3.91 ± 0.29	0.000
	Baseline	0.04 ± 0.30	0.00 ± 0.00	0.320	0.04 ± 0.30	0.00 ± 0.00	0.320	0.04 ± 0.30	0.00 ± 0.00	0.320
Undesirable hair changes	3rd cycle	0.04 ± 0.30	0.00 ± 0.00	1.000	0.04 ± 0.30	0.00 ± 0.00	1.000	0.04 ± 0.30	0.00 ± 0.00	1.000
	6th cycle	0.04 ± 0.30	0.00 ± 0.00	1.000	0.04 ± 0.30	0.00 ± 0.00	1.000	0.04 ± 0.30	0.00 ± 0.00	1.000

DSG: desogestrel and DRSP: drospirenone.

**Table 5:** The results of group and subgroup analyses according to the comparison of mean WHAQ scores changes within groups.

	Within group												
Repeated ANOVA													
		Premenstrual				Menstrual				Postmenstrual			
Mean WHAQ scores*		DSG (±SE)	Sig.	DRSP (±SE)	Sig.	DSG (±SE)	Sig.	DRSP (±SE)	Sig.	DSG (±SE)	Sig.	DRSP (±SE)	Sig.
	Baseline–3rd cycle	0.42 ± 0.41	0.307	6.56 ± 0.79	0.000	−0.69 ± 0.34	0.046	4.44 ± 0.76	0.000	−0.11 ± 0.26	0.672	1.36 ± 0.34	0.000
Total	Baseline–6th cycle	3.24 ± 0.77	0.000	13.71 ± 1.25	0.000	1.73 ± 0.63	0.008	9.93 ± 0.96	0.000	0.47 ± 0.30	0.132	1.87 ± 0.45	0.000
	3rd cycle–6th cycle	2.82 ± 0.65	0.000	7.16 ± 0.91	0.000	2.42 ± 0.58	0.000	5.49 ± 0.55	0.000	0.58 ± 0.27	0.041	0.51 ± 0.25	0.050
	Baseline–3rd cycle	0.42 ± 0.19	0.031	2.31 ± 0.45	0.000	0.04 ± 0.17	0.800	1.58 ± 0.45	0.001	0.04 ± 0.13	0.736	0.58 ± 0.21	0.008
Negative effect	Baseline–6th cycle	2.16 ± 0.44	0.000	5.82 ± 0.58	0.000	1.49 ± 0.33	0.000	4.36 ± 0.50	0.000	0.38 ± 0.18	0.039	1.31 ± 0.23	0.000
	3rd cycle–6th cycle	1.73 ± 0.40	0.000	3.51 ± 0.43	0.000	1.44 ± 0.30	0.000	2.78 ± 0.39	0.000	0.33 ± 0.15	0.034	0.73 ± 0.18	0.000
	Baseline–3rd cycle	0.20 ± 0.13	0.130	1.87 ± 0.27	0.000	−0.64 ± 0.17	0.000	1.20 ± 0.31	0.000	−0.20 ± 0.12	0.107	0.40 ± 0.14	0.005
Water retention	Baseline–6th cycle	0.844 ± 0.32	0.012	3.53 ± 0.46	0.000	−0.36 ± 0.37	0.345	2.29 ± 0.39	0.000	−0.02 ± 0.14	0.872	0.53 ± 0.18	0.005
	3rd cycle–6th cycle	0.644 ± 0.31	0.040	1.67 ± 0.32	0.000	0.29 ± 0.31	0.365	1.09 ± 0.25	0.000	0.18 ± 0.12	0.132	0.13 ± 0.11	0.225
	Baseline–3rd cycle	0.38 ± 0.18	0.036	2.58 ± 0.38	0.000	0.60 ± 0.21	0.007	2.24 ± 0.40	0.000	0.11 ± 0.14	0.417	0.51 ± 0.18	0.006
Impaired concentration	Baseline–6th cycle	1.22 ± 0.34	0.001	4.91 ± 0.63	0.000	1.36 ± 0.29	0.000	4.16 ± 0.52	0.000	0.36 ± 0.17	0.041	0.82 ± 0.18	0.000
	3rd cycle–6th cycle	0.84 ± 0.28	0.005	2.33 ± 0.46	0.000	0.76 ± 0.23	0.002	1.91 ± 0.29	0.000	0.24 ± 0.16	0.140	0.31 ± 0.11	0.009
	Baseline–3rd cycle	−0.11 ± 0.14	0.417	0.76 ± 0.12	0.000	−0.04 ± 0.10	0.643	0.69 ± 0.14	0.000	−0.18 ± 0.11	0.103	0.16 ± 0.08	0.051
Increased appetite	Baseline–6th cycle	−0.20 ± 0.18	0.284	1.02 ± 0.18	0.000	0.16 ± 0.18	0.399	1.18 ± 0.18	0.000	−0.07 ± 0.12	0.570	0.16 ± 0.10	0.128
	3rd cycle–6th cycle	−0.09 ± 0.13	0.486	0.27 ± 0.13	0.050	0.20 ± 0.14	0.162	0.49 ± 0.14	0.001	0.11 ± 0.09	0.229	0.00 ± 0.08	1.00
	Baseline–3rd cycle	−0.47 ± 0.12	0.000	−0.96 ± 0.12	0.000	−0.58 ± 0.12	0.000	−1.27 ± 0.16	0.000	0.11 ± 0.12	0.375	−0.29 ± 0.15	0.068
Feeling of well being	Baseline–6th cycle	−0.78 ± 0.13	0.000	−1.58 ± 0.13	0.000	−0.84 ± 0.16	0.000	−1.98 ± 0.16	0.000	−0.18 ± 0.15	0.242	−0.96 ± 0.11	0.000
	3rd cycle–6th cycle	−0.31 ± 0.12	0.015	−0.62 ± 0.12	0.000	−0.27 ± 0.13	0.038	−0.71 ± 0.17	0.000	−0.29 ± 0.15	0.063	−0.67 ± 0.14	0.000
	Baseline–3rd cycle	0.00 ± 0.00	—	0.00 ± 0.00	—	0.00 ± 0.00	—	0.00 ± 0.00	—	0.00 ± 0.00	—	0.00 ± 0.00	—
Undesirable hair changes	Baseline–6th cycle	0.00 ± 0.00	—	0.00 ± 0.00	—	0.00 ± 0.00	—	0.00 ± 0.00	—	0.00 ± 0.00	—	0.00 ± 0.00	—
	3rd cycle–6th cycle	0.00 ± 0.00	—	0.00 ± 0.00	—	0.00 ± 0.00	—	0.00 ± 0.00	—	0.00 ± 0.00	—	0.00 ± 0.00	—

DSG: desogestrel and DRSP: drospirenone.

*Mean WHAQ scores were calculated from summed scores of all the subjects divided by the number of subjects.

**Table 6:** The comparison of the prevalence of adverse effects (episodes per 100 samples) between both groups.

Adverse events (AEs)	Prevalence of events (%)		Between-group *P* value
	DSG*	DRSP*
Nausea/vomiting	8 (17.8)	1 (2.2)	NS
Dizziness	1 (2.2)	4 (8.9)	NS
Amenorrhea	3 (6.7)	1 (2.2)	NS
Spotting	2 (4.4)	2 (4.4)	NS
Mastalgia	1 (2.2)	0 (0)	NS

*DSG: desogestrel group and DRSP: drospirenone group.

**Table 7:** The comparison of the body-weight change between both groups.

Body weight (kg)	Comparison		Between-group *P* value
	DSG* (*n* = 45)	DRSP* (*n* = 45)
Baseline	55.6 (±7.9)	56.5 (±8.4)	0.446
3rd cycle	55.9 (±8.1)	56.3 (±8.5)	0.232
6th cycle	55.9 (±7.9)	55.5 (±8.7)	0.003

*DSG: desogestrel group and DRSP: drospirenone group.
